# Bis(acetonitrile-κ*N*)diaqua­bis­(perchlorato-κ*O*)copper(II)

**DOI:** 10.1107/S1600536811027309

**Published:** 2011-07-23

**Authors:** Viktor A. Tafeenko, Stanislav I. Gurskiy, Leonid A. Aslanov

**Affiliations:** a Chemistry Department, Moscow State University, 119991 Moscow, Russian Federation

## Abstract

In the title compound, [Cu(ClO_4_)_2_(CH_3_CN)_2_(H_2_O)_2_], the Cu^2+^ ion, located on a special position (site symmetry 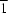), is coordinated by six monodentate ligands, *viz.* an *N*-coordin­ated acetonitrile, a perchlorate anion and a water mol­ecule, and their symmetry-related counterparts. The perchlorate anion is disordered over two sets of sites with occupancies of 0.53 (2) and 0.47 (2). The crystal structure is stabilized by O—H⋯O hydrogen bonds involving the perchlorate ion and aqua H atoms.

## Related literature

For details of the changing Cu(II/I) redox potential with increasing acetonitrile contents in water–acetonitrile solution, see: Cox *et al.* (1988 ▶); Verma & Sood (1979 ▶); Sumalekshmy & Gopidas (2005 ▶); Ajayakumar *et al.* (2009 ▶); Drew *et al.* (1985 ▶). For the dependence of the luminescent properties (emission energy) of the 3-cyano-4-dicyano­methyl­ene-5-oxo-4,5-dihydro-1*H*-pyrrol-2-olate (*A*)-based salts depend on the mol­ecular environment around (*A*), see: Tafeenko *et al.* (2009 ▶, 2010 ▶). For transition metals as fluorescence quenchers, see: Xu *et al.* (2005 ▶, 2010 ▶). For a previous study on the formation of related compounds, see: Inamo *et al.* (2001 ▶).  
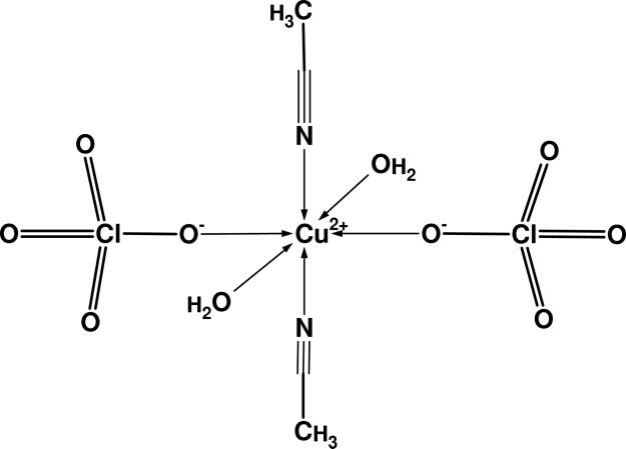


## Experimental

### 

#### Crystal data

[Cu(ClO_4_)_2_(C_2_H_3_N)_2_(H_2_O)_2_]
*M* *_r_* = 380.59
Triclinic, 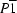

*a* = 5.581 (1) Å
*b* = 7.244 (2) Å
*c* = 8.733 (2) Å
α = 82.82 (2)°
β = 76.86 (1)°
γ = 77.12 (1)°
*V* = 334.1 (1) Å^3^
*Z* = 1
Ag *K*α radiation
λ = 0.56085 Å
μ = 1.09 mm^−1^
*T* = 296 K
0.15 × 0.1 × 0.08 mm

#### Data collection

Enraf–Nonius CAD-4 diffractometer
2518 measured reflections
1259 independent reflections
1020 reflections with *I* > 2s(*I*)
*R*_int_ = 0.049
2 standard reflections every 120 min  intensity decay: none

#### Refinement

*R*[*F* ^2^ > 2σ(*F* ^2^)] = 0.066
*wR*(*F* ^2^) = 0.192
*S* = 1.08
1259 reflections
138 parameters
11 restraints
H atoms treated by a mixture of independent and constrained refinement
Δρ_max_ = 0.70 e Å^−3^
Δρ_min_ = −1.09 e Å^−3^

### 

Data collection: *CAD-4 Software* (Enraf–Nonius, 1989 ▶); cell refinement: *CAD-4 Software*; data reduction: *XCAD4* (Harms & Wocadlo, 1995 ▶); program(s) used to solve structure: *SHELXS97* (Sheldrick, 2008 ▶); program(s) used to refine structure: *SHELXL97* (Sheldrick, 2008 ▶); molecular graphics: *DIAMOND* (Brandenburg, 2000 ▶); software used to prepare material for publication: *WinGX* (Farrugia, 1999 ▶).

## Supplementary Material

Crystal structure: contains datablock(s) I, global. DOI: 10.1107/S1600536811027309/fi2110sup1.cif

Structure factors: contains datablock(s) I. DOI: 10.1107/S1600536811027309/fi2110Isup2.hkl

Additional supplementary materials:  crystallographic information; 3D view; checkCIF report

## Figures and Tables

**Table 1:** Hydrogen-bond geometry (Å, °)

*D*—H⋯*A*	*D*—H	H⋯*A*	*D*⋯*A*	*D*—H⋯*A*
O1—H11⋯O3^i^	0.83 (7)	2.03 (7)	2.758 (17)	146 (6)
O1—H12⋯O3^ii^	0.79 (9)	2.22 (10)	3.00 (3)	165 (8)

Symmetry codes: (i) 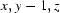; (ii) 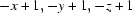.

**Fig. 1.**
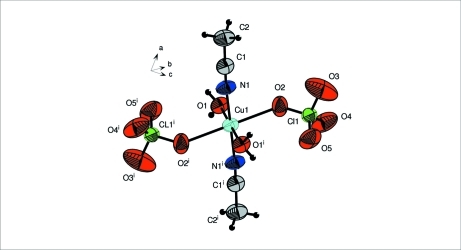
The molecular structure of the title compound, with displacement ellipsoids drawn at the 50% probability level. Only one of the two disordered positions of the perchlorate ion is shown. Symmetry code: (i) – x,1 – y, – z.

**Fig. 2.**
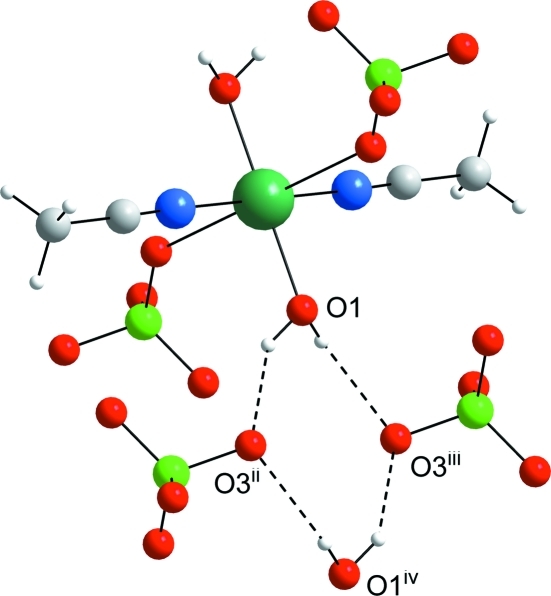
The hydrogen bonding pattern in the title compound. H bonds are drawn as dashed lines. Symmetry codes (ii) x, 1–y, z; (iii): 1–x, 1–y, 1–z; (iv) 1–x, –y, 1–z.

## supplementary crystallographic information

### Comment

It was found (Tafeenko *et al.*, 2009; Tafeenko *et al.*, 2010) that the luminescent properties (emission energy) of the 3-cyano-4-dicyanomethylene-5-oxo-4,5-dihydro-1*H*-pyrrol-2-olate (A)-based salts depend on the molecular environment around (A). To investigate the effect of transition metals, which act usually as fluorescence quenchers (Xu *et al.*, 2005, 2010), we made an attempt to synthesize (A)-based salts with Cu^2+^. Acetonitrile treatment of crude crystalline mass, obtained after evaporation of CuSO_4_ + BaA_2_ (equivalent amounts) mixture in water-ethanol (*v*/*v*=1/1) solution, and posterior evaporation of an acetonitrile solution in an air atmosphere at room temperature resulted in formation of (A)-based salts with Cu^+^. We saw several reasons for the reduction of Cu^2+^ to Cu^+^:

- iodide anion I^-^ reduces Cu^2+^ in water-ethanol solution, as it was detected that BaA_2_ salt used for synthesis contained I^-^;

- anion (A) exhibits of reducing properties;

- acetonitrile-water mixture causes reduction of copper(II) to copper(I), as it was documented that i) copper(II) salt solution in acetonitrile-water mixture is a powerful oxidizer of organic molecules (Verma *et al.*, 1979; Cox *et al.*,1988; Sumalekshmy *et al.*, 2005; Ajayakumar *et al.*, 2009); ii) acetonitrile can reduce Cu^2+^ to Cu^+^ (Drew *et al.*, 1985).

To find out whether acetonitrile-water mixture can cause a reduction of Cu^2+^ to Cu^+^, we prepared a Cu(ClO_4_)_2_ solution in a mixture of 99.5% CH_3_CN and 0.5% H_2_O (volume percentage) and evaporated this solution, which yielded crystals of the title compound, [Cu(CH_3_CN)_2_(H_2_O)_2_(ClO_4_)_2_]. No other phases could be detected using powder-XRD. The crystal and molecular structure of the title compound (Fig.1) is presented in this paper.

The structure is composed of monomeric units built up around a Cu^2+^ on a special position (site symmetry –1). The Cu^2+^ cation is surrounded by six monodentate ligands, *viz.* an N–coordinated acetonitrile, a perchlorate anion and a water molecule, and their symmetry related counterparts. The perchlorate anion is disordered over two positions, with occupancies (0.53 (2) and 0.47 (2)), but it's O atoms displacement ellipsoids are still quite large, indicating possible rotational disorder, with the rotation axis passing through oxygen O1 of the perchlorate coordinated with Cu1 and Cl1. The complex adopts an elongated octahedral coordination geometry.

The axial Cu–O1 (perchlorate) bond length is 2.401 (15) and in plane Cu–N1(acetonitrile) 1.960 (5), Cu1–O2(aqua) 1.950 (5) Å respectively.

Besides ionic forces, the crystal structure is stabilized by hydrogen bonding interaction *via* the perchlorate and aqua H atoms (Fig. 2).

In conclusion, we have to note that the structure of the title compound differs from reported by Inamo with co-workers (Inamo *et al.*, 2001) compounds. They reporteded the formation of [Cu(H_2_O)_n_(CH_3_CN)_(6-n)_]^2+^ (n = 0–3) in (water/acetonitrile) solutions with [Cu(H_2_O)_m_(CH_3_CN)_6-m_]^2+^ (m < 3) as the dominant species for H_2_O concentration lower than 0.5 *M* (99% acetonitrile).

### Experimental

Blue crystals of the title salt were obtained by slow evaporation of 0.045 *M* Cu(H_2_O)_6_(ClO_4_)_2_ solution in acetonitrile at room temperature in an air atmosphere. The crystals are not stable in the open air, so suitable for X-ray investigation crystal was placed in a sealed capillary. Copper(II) perchlorate hexahydrate of 98% (Aldrich) grade was used for synthesis. Acetonitrile was boiled with phosphorus pentaoxide and then distilled at 353 K.

### Refinement

During the refinement a difference maps showed peaks consistent with the perchlorate atoms Cl1,O2—O5 being unequally disorder over two interpenetrating sites. This was allowed for by use of the appropriate *SHELXL* SAME, EADP restraints. At convergence the perchlorate disorder was modelled with occupancies (0.53 (2) and 0.47 (2)).

The positions of the H atoms of the water molecule were determined from Fourier difference maps and refined freely; the positions of the H atoms of the methyl group were placed in calculated positions and allowed to ride on their parent atoms [C—H = 0.96 Å]. *U*_iso_(H) = *xU*_eq_(parent atom), where *x* = 1.5 for attached C atoms.

### Crystal data

**Table:**

[Cu(ClO_4_)_2_(C_2_H_3_N)_2_(H_2_O)_2_]	*Z* = 1
*M**_r_* = 380.59	*F*(000) = 191
Triclinic, *P*1	*D*_x_ = 1.891 Mg m^−^^3^
Hall symbol: -P 1	Melting point: 422 K
*a* = 5.581 (1) Å	Ag *K*α radiation, λ = 0.56085 Å
*b* = 7.244 (2) Å	Cell parameters from 25 reflections
*c* = 8.733 (2) Å	θ = 11–13°
α = 82.82 (2)°	µ = 1.09 mm^−^^1^
β = 76.86 (1)°	*T* = 296 K
γ = 77.12 (1)°	Prism, light-blue
*V* = 334.1 (1) Å^3^	0.15 × 0.1 × 0.08 mm

### Data collection

**Table:**

Enraf–Nonius CAD-4 diffractometer	*R*_int_ = 0.049
Radiation source: fine-focus sealed tube	θ_max_ = 20.0°, θ_min_ = 1.9°
graphite	*h* = −6→6
non–profiled ω scans	*k* = −8→8
2518 measured reflections	*l* = −10→10
1259 independent reflections	2 standard reflections every 120 min
1020 reflections with *I* > 2s(*I*)	intensity decay: none

### Refinement

**Table:**

Refinement on *F*^2^	Primary atom site location: structure-invariant direct methods
Least-squares matrix: full	Secondary atom site location: difference Fourier map
*R*[*F*^2^ > 2σ(*F*^2^)] = 0.066	Hydrogen site location: inferred from neighbouring sites
*wR*(*F*^2^) = 0.192	H atoms treated by a mixture of independent and constrained refinement
*S* = 1.08	*w* = 1/[σ^2^(*F*_o_^2^) + (0.1161*P*)^2^ + 0.4339*P*] where *P* = (*F*_o_^2^ + 2*F*_c_^2^)/3
1259 reflections	(Δ/σ)_max_ = 0.042
138 parameters	Δρ_max_ = 0.70 e Å^−^^3^
11 restraints	Δρ_min_ = −1.09 e Å^−^^3^

### Special details

**Table:**

Geometry. All e.s.d.'s (except the e.s.d. in the dihedral angle between two l.s. planes) are estimated using the full covariance matrix. The cell e.s.d.'s are taken into account individually in the estimation of e.s.d.'s in distances, angles and torsion angles; correlations between e.s.d.'s in cell parameters are only used when they are defined by crystal symmetry. An approximate (isotropic) treatment of cell e.s.d.'s is used for estimating e.s.d.'s involving l.s. planes.
Refinement. Refinement of *F*^2^ against ALL reflections. The weighted *R*-factor *wR* and goodness of fit *S* are based on *F*^2^, conventional *R*-factors *R* are based on *F*, with *F* set to zero for negative *F*^2^. The threshold expression of *F*^2^ > σ(*F*^2^) is used only for calculating *R*-factors(gt) *etc*. and is not relevant to the choice of reflections for refinement. *R*-factors based on *F*^2^ are statistically about twice as large as those based on *F*, and *R*- factors based on ALL data will be even larger.

### Fractional atomic coordinates and isotropic or equivalent isotropic displacement parameters (Å^2^)

**Table:**

	*x*	*y*	*z*	*U*_iso_*/*U*_eq_	Occ. (<1)
Cu1	0.0000	0.5000	0.5000	0.0477 (4)	
O1	0.2556 (10)	0.2741 (6)	0.5295 (6)	0.0574 (12)	
N1	0.2362 (12)	0.5987 (8)	0.3237 (7)	0.0631 (16)	
C1	0.3717 (13)	0.6605 (9)	0.2302 (8)	0.0555 (15)	
C2	0.5546 (16)	0.7387 (12)	0.1089 (9)	0.072 (2)	
H2A	0.7197	0.6899	0.1297	0.109*	
H2B	0.5174	0.8747	0.1088	0.109*	
H2C	0.5478	0.7034	0.0079	0.109*	
Cl1	0.120 (3)	0.803 (2)	0.7550 (16)	0.0538 (10)	0.53 (2)
O2	0.195 (3)	0.656 (3)	0.651 (2)	0.082 (6)	0.53 (2)
O3	0.295 (5)	0.921 (3)	0.694 (3)	0.142 (9)	0.53 (2)
O4	0.143 (3)	0.729 (3)	0.9072 (14)	0.105 (6)	0.53 (2)
O5	−0.124 (3)	0.902 (2)	0.755 (2)	0.096 (5)	0.53 (2)
Cl11	0.132 (3)	0.820 (2)	0.7453 (18)	0.0538 (10)	0.47 (2)
O21	0.112 (5)	0.637 (2)	0.717 (2)	0.079 (6)	0.47 (2)
O31	0.383 (2)	0.837 (3)	0.729 (2)	0.080 (5)	0.47 (2)
O41	0.008 (6)	0.853 (6)	0.899 (3)	0.22 (2)	0.47 (2)
O51	0.017 (6)	0.955 (2)	0.644 (4)	0.156 (12)	0.47 (2)
H11	0.212 (12)	0.172 (10)	0.565 (7)	0.041 (16)*	
H12	0.357 (17)	0.228 (12)	0.458 (10)	0.07 (3)*	

### Atomic displacement parameters (Å^2^)

**Table:**

	*U*^11^	*U*^22^	*U*^33^	*U*^12^	*U*^13^	*U*^23^
Cu1	0.0492 (7)	0.0344 (6)	0.0530 (7)	−0.0110 (4)	0.0021 (4)	0.0024 (4)
O1	0.063 (3)	0.034 (2)	0.064 (3)	−0.004 (2)	0.001 (2)	0.002 (2)
N1	0.060 (3)	0.045 (3)	0.068 (3)	−0.013 (3)	0.014 (3)	0.008 (2)
C1	0.057 (4)	0.042 (3)	0.060 (4)	−0.006 (3)	−0.005 (3)	0.002 (3)
C2	0.073 (5)	0.075 (5)	0.060 (4)	−0.028 (4)	0.005 (4)	0.016 (3)
Cl1	0.0544 (13)	0.040 (2)	0.0630 (16)	−0.0150 (12)	0.0034 (11)	−0.0097 (13)
O2	0.062 (9)	0.085 (11)	0.107 (13)	0.011 (7)	−0.033 (9)	−0.051 (10)
O3	0.115 (16)	0.087 (11)	0.23 (2)	−0.061 (11)	−0.042 (14)	0.062 (13)
O4	0.093 (11)	0.152 (15)	0.046 (6)	−0.002 (9)	0.000 (6)	0.013 (7)
O5	0.072 (8)	0.088 (11)	0.110 (11)	0.018 (7)	−0.007 (7)	−0.028 (8)
Cl11	0.0544 (13)	0.040 (2)	0.0630 (16)	−0.0150 (12)	0.0034 (11)	−0.0097 (13)
O21	0.111 (17)	0.047 (7)	0.090 (12)	−0.027 (9)	−0.039 (10)	0.003 (7)
O31	0.044 (7)	0.117 (14)	0.085 (9)	−0.034 (8)	0.006 (6)	−0.028 (9)
O41	0.18 (3)	0.31 (4)	0.17 (3)	−0.16 (3)	0.12 (2)	−0.15 (3)
O51	0.19 (3)	0.041 (8)	0.26 (3)	−0.028 (10)	−0.11 (2)	0.040 (11)

### Geometric parameters (Å, °)

**Table:**

Cu1—O1	1.950 (5)	C2—H2C	0.9600
Cu1—N1	1.960 (5)	Cl1—O5	1.390 (14)
Cu1—O2	2.401 (15)	Cl1—O4	1.391 (14)
O1—H11	0.83 (7)	Cl1—O3	1.414 (14)
O1—H12	0.79 (9)	Cl1—O2	1.418 (12)
N1—C1	1.103 (9)	Cl11—O51	1.386 (15)
C1—C2	1.450 (9)	Cl11—O41	1.387 (15)
C2—H2A	0.9600	Cl11—O31	1.409 (14)
C2—H2B	0.9600	Cl11—O21	1.413 (13)
			
O1—Cu1—O1^i^	180.000 (1)	C1—C2—H2C	109.5
O1—Cu1—N1	90.4 (2)	H2A—C2—H2C	109.5
O1^i^—Cu1—N1	89.6 (2)	H2B—C2—H2C	109.5
N1—Cu1—N1^i^	180.0 (3)	O5—Cl1—O4	110.2 (13)
O1—Cu1—O2^i^	93.3 (4)	O5—Cl1—O3	110.9 (15)
N1—Cu1—O2^i^	97.7 (6)	O4—Cl1—O3	110.2 (14)
N1^i^—Cu1—O2^i^	82.3 (6)	O5—Cl1—O2	112.1 (11)
O1—Cu1—O2	86.7 (4)	O4—Cl1—O2	110.3 (13)
O2^i^—Cu1—O2	180.0 (7)	O3—Cl1—O2	102.9 (14)
Cu1—O1—H11	120 (4)	Cl1—O2—Cu1	137.3 (11)
Cu1—O1—H12	123 (6)	O51—Cl11—O41	109 (2)
H11—O1—H12	93 (7)	O51—Cl11—O31	109.9 (15)
C1—N1—Cu1	176.0 (7)	O41—Cl11—O31	108.2 (15)
N1—C1—C2	178.6 (8)	O51—Cl11—O21	109.8 (13)
C1—C2—H2A	109.5	O41—Cl11—O21	107.7 (16)
C1—C2—H2B	109.5	O31—Cl11—O21	112.3 (14)
H2A—C2—H2B	109.5		

Symmetry codes: (i) −*x*, −*y*+1, −*z*+1.

### Hydrogen-bond geometry (Å, °)

**Table:**

*D*—H···*A*	*D*—H	H···*A*	*D*···*A*	*D*—H···*A*
O1—H11···O3^ii^	0.83 (7)	2.03 (7)	2.758 (17)	146 (6)
O1—H12···O3^iii^	0.79 (9)	2.22 (10)	3.00 (3)	165 (8)

Symmetry codes: (ii) *x*, *y*−1, *z*; (iii) −*x*+1, −*y*+1, −*z*+1.
